# Circulating microRNA: Myocardium-derived prenatal biomarker of ventricular septal defects

**DOI:** 10.3389/fgene.2022.899034

**Published:** 2022-08-11

**Authors:** Yiru Yang, Hainan Yang, Xihua Lian, Shuping Yang, Haolin Shen, Shufen Wu, Xiali Wang, Guorong Lyu

**Affiliations:** ^1^ Department of Ultrasound, The Second Affiliated Hospital of Fujian Medical University, Quanzhou, Fujian, China; ^2^ Department of Ultrasound, The First Affiliated Hospital of Xiamen University, Xiamen, Fujian, China; ^3^ Department of Pathology and Biomedical Science, University of Otago, Christchurch, New Zealand; ^4^ Department of Ultrasound, Zhangzhou Affiliated Hospital of Fujian Medical University, Zhangzhou, Fujian, China; ^5^ Collaborative Innovation Center for Maternal and Infant Health Service Application Technology, Quanzhou Medical College, Quanzhou, Fujian, China

**Keywords:** ventricular septal defect (VSD), circulating microRNA (miRNA), prenatal diagnosis (MeSH), biomarker, fetus [mesh]

## Abstract

**Background:** Recently, circulating microRNAs (miRNAs) from maternal blood and amniotic fluid have been used as biomarkers for ventricular septal defect (VSD) diagnosis. However, whether circulating miRNAs are associated with fetal myocardium remains unknown.

**Methods:** Dimethadione (DMO) induced a VSD rat model. The miRNA expression profiles of the myocardium, amniotic fluid and maternal serum were analyzed. Differentially expressed microRNAs (DE-microRNAs) were verified by qRT–PCR. The target gene of miR-1-3p was confirmed by dual luciferase reporter assays. Expression of amniotic fluid-derived DE-microRNAs was verified in clinical samples.

**Results:** MiRNAs were differentially expressed in VSD fetal rats and might be involved in cardiomyocyte differentiation and apoptosis. MiR-1-3p, miR-1b and miR-293-5p were downregulated in the myocardium and upregulated in amniotic fluid/maternal serum. The expression of amniotic fluid-derived DE-microRNAs (miR-1-3p, miR-206 and miR-184) was verified in clinical samples. Dual luciferase reporter assays confirmed that miR-1-3p directly targeted SLC8A1/NCX1.

**Conclusion:** MiR-1-3p, miR-1b and miR-293-5p are downregulated in VSD myocardium and upregulated in circulation and may be released into circulation by cardiomyocytes. MiR-1-3p targets SLC8A1/NCX1 and participates in myocardial apoptosis. MiR-1-3p upregulation in circulation is a direct and powerful indicator of fetal VSD and is expected to serve as a prenatal VSD diagnostic marker.

## 1 Introduction

Ventricular septal defects (VSDs) are the most common congenital heart defects (CHDs), accounting for approximately 40% of CHDs (Spicer et al., 2014; Cox et al., 2020). Accurate prenatal diagnosis is helpful for reducing mortality and improving prognosis (van Nisselrooij et al., 2020). However, it was reported that due to factors such as the experience of sonographers and the quality of ultrasound images, approximately half of CHDs were not identified prenatally. Even when the ultrasound image quality is good, 31% of CHD cases are still missed (van Nisselrooij et al., 2020). Therefore, it is of great significance to seek biomarkers for the prenatal diagnosis of VSD to improve the accuracy of diagnosis and pregnancy outcomes.

Human genetics research has identified many genes related to hereditary and sporadic CHD that encode transcription factors that regulate the morphogenesis of the ventricular septum or outflow tract during heart development (Bruneau, 2008). Epigenetic mechanisms, including DNA methylation and noncoding RNA (ncRNA), are involved in the pathogenesis and phenotype of VSD (Grunert et al., 2016; Thomford et al., 2018; Yang et al., 2021). MicroRNAs (miRNAs), as highly conserved ncRNAs, regulate gene expression at the posttranscriptional level by binding to target genes, inducing epigenetic modifications, which are closely related to the cell cycle and mammalian development (Pulignani and Andreassi, 2019; Panni et al., 2020). By regulating VSD-related transcription factors, miRNAs participate in the proliferation and differentiation of cardiomyocytes, and morphogenesis of heart, as well as pathophysiological processes such as myocardial hypoxia and cardiac remodelling, which are interrelated with the occurrence, progression and outcome of VSD (Smith et al., 2015; Islas and Moreno-Cuevas, 2018; Sabour et al., 2018; Meng et al., 2020). MiRNAs can be encapsulated in lipid vesicles or connected with protein or lipoprotein complexes to ensure stability and avoid degradation during extracellular secretion. Therefore, miRNAs have the potential to be diagnostic markers of diseases (Creemers et al., 2012; Mori et al., 2019).

Maternal blood is commonly used for prenatal diagnosis and is easy to obtain, while amniotic fluid has a high content of fetal free nucleic acids and is less likely to be contaminated by maternal nucleic acids (Hui et al., 2012). In recent years, people have attempted to use circulating miRNAs derived from maternal blood and amniotic fluid as biomarkers for CHD diagnosis (Song et al., 2018; Jin et al., 2021; Yang et al., 2021). It is, however, unclear whether circulating miRNAs are associated with fetal heart tissue. Thus, we analyzed the expression profiles of amniotic fluid-derived and maternal serum-derived miRNAs in fetal VSD rats to explore the relationship between myocardium-derived and circulating miRNAs. Furthermore, the possibility of using circulating miRNAs as VSD prenatal biomarkers was verified in the clinical samples to serve as the basis for circulating miRNAs to assist prenatal diagnosis.

## 2 Materials and methods

### 2.1 Construction of a ventricular septal defect rat model

Sprague–Dawley (SD) rats (Shanghai SLAC Laboratory Animal Co., Ltd.) were selected to construct VSD models. Female and male rats in estrus were kept in cages at a ratio of 2:1. The day when vaginal plugs were found was recorded as embryonic day 0 (D0). The dams were separated from male rats and randomized to a negative control (NC) group and a dimethadione (DMO) group. The mean maternal age of rats was 14.44 (SD, 5.17) weeks vs. 15.44 (SD, 4.06) weeks in the NC and DMO groups, respectively.

From 19:00 on D8, the DMO group was given 5 ml/kg DMO (drug concentration, 60 mg/ml) by oral gavage once every 12 h six times, while the NC group was given the same dose of distilled water at the same time. The rats were fed standard food and distilled water *ad libitum* and received humane care.

**FIGURE 1:**
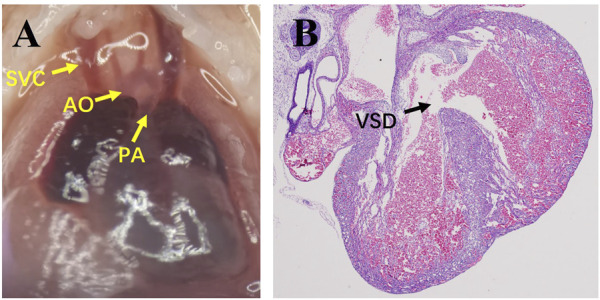
Images of microdissection and the pathology of VSD fetuses. **(A)**, Microdissection shows the correct connection of major vessels; **(B)**, pathology shows VSD. SVC, superior vena cava; AO, aorta; PA, pulmonary artery; VSD, ventricular septal defect.

In the DMO group, excluding fetuses with umbilical hernia, subcutaneous edema or other obvious abnormal appearances, VSD fetuses without malformation of major vessels were selected as the VSD group (Figure 1), and fetuses with intact ventricular septum were selected as the non-VSD group.

### 2.2 Sample collection and pretreatment

On D19, the pregnant mice were anaesthetized by intraperitoneal injection of pentobarbital (40 mg/kg), and the amniotic fluid was carefully collected with a sterile syringe after the uterus was exposed. The amniotic fluid was immediately centrifuged at 1,200 g and 4°C for 10 min, and the supernatant was recovered and stored at −80°C.

Blood was withdrawn through cardiac puncture and incubated at room temperature for 1 h. The coagulated blood was centrifuged twice at 4°C (1700 g, 10 min and 2000 g, 10 min), and then the supernatant was stored at −80°C.

The fetus was obtained by caesarean section. Microdissection was used to observe the position and connection of the major blood vessels. After that, the fetal heart was removed and washed in cold PBS solution. The heart tissue was embedded in paraffin according to the routine procedure. Parts of the wax blocks were cut into 3 μm slices and prepared for HE staining to observe the ventricular septum, and other parts were stored for RNA extraction.

### 2.3 Collection of clinical amniotic fluid samples

From August 2020 to June 2021, women who visited the Second Affiliated Hospital of Fujian Medical University were included when ultrasound-guided amniocentesis met their clinical needs. The amniotic fluid was extracted after informed consent was obtained from all pregnant women. The amniotic fluid of fetuses with a normal chromosome karyotype and without pregnancy complications or other diseases that may affect the growth and development of the fetus was collected. According to the results of follow-up after birth or induction of labor, they were classified into the VSD group and the NC group. General characteristics of pregnant women are displayed in Table 1. This study was carried out in accordance with The Code of Ethics of the World Medical Association (Declaration of Helsinki) and was approved by the Medical Ethics Committee of the Second Affiliated Hospital of Fujian Medical University (2019-233, 2021-73).

**TABLE 1:** General characteristics of pregnant women.

	NC group (*n* = 7)	VSD group (*n* = 7)	p
Maternal age (years)	30.00 ± 4.90	29.00 ± 5.03	0.713
Gestational age (weeks)	21.80 ± 3.12	24.59 ± 1.35	0.140
Weight (kg)	53.04 ± 7.18	56.92 ± 8.67	0.397
Parity(n)	0.00 (0.00,2.00)	0.00 (0.00,2.00)	1.000
Oligohydramnios or polyhydramnios (%)	0.00	0.00	—

NC, negative control; VSD, ventricular septal defect.

### 2.4 MicroRNA sequencing

Corresponding kits were used to extract total RNA/miRNA from wax blocks of the myocardium (RecoverAll™ Total Nucleic Acid Isolation Kit, Ambion, Thermo Fisher Scientific), amniotic fluid (HiPure Universal RNA Mini Kit, Magen) and maternal serum (miRNeasy Mini Kit, QIAGEN). A cDNA library of amniotic fluid-derived miRNAs (QIAseq miRNA Library Kit, QIAGEN) and myocardium-derived and serum-derived miRNAs (TruSeq Small RNA Library Preparation kit, Illumina) was constructed, respectively. The library preparations were sequenced on the Illumina HiSeq 2500 sequencing system (for myocardium-derived and serum-derived miRNAs) and Illumina NovaSeq 6000 system (for amniotic fluid-derived miRNAs).

### 2.5 Sequencing data processing

Bcl2fastq (bcl2fastq, RRID:SCR_015058) was used to perform recognition on the original image and convert it into the original sequence. The Fastx-toolkit (FASTX-Toolkit, RRID:SCR_005534) was used to evaluate and filter the quality. The expression was normalized using the transcripts read number per million (TPM) and counts per million (CPM) method. DESeq (DESeq, RRID:SCR_000154) and an R package (LIMMA, RRID:SCR_010943) was used to perform differential expression analysis. The threshold values *p* < 0.05 and fold change (FC) ≥ 2 indicated upregulated differentially expressed microRNA (DE-microRNA), while *p* < 0.05 and FC ≤ 0.5 indicated downregulated DE-microRNA.

### 2.6 Target gene prediction and bioinformatics analysis of differentially expressed microRNAs

MiRWalk 3.0 (miRWalk, RRID:SCR_016509) predicted the target genes of DE-microRNAs. Gene Ontology (GO) and Kyoto Encyclopedia of Genes and Genomes (KEGG) analyzes of target genes whose binding probability > 0.95 were performed by g:Profiler (version e104_eg51_p15_3922dba). String (https://cn.string-db.org/) was used to perform protein–protein interaction (PPI) analysis and establish PPI networks. The plug-in “cytoHubba” of Cytoscape (Cytoscape, RRID:SCR_003032) screened out the top 20 hub genes in the PPI networks.

### 2.7 Quantitative real-time polymerase chain reaction

Total RNA was extracted by TRIzol (Invitrogen, Thermo Fisher Scientific) and the RecoverAll™ Total Nucleic Acid Isolation Kit (Ambion, Thermo Fisher Scientific) from amniotic fluid, serum and myocardium wax blocks. MiRNA First Strand cDNA Synthesis (Sangon Biotech) was used to reverse transcribe RNA to cDNA. Polymerase chain reaction (PCR) was performed according to the manual of the TB Green Premix Ex Taq kit (TAKARA). All reactions were performed in triplicate. The result was normalized to U6 (Universal U6 Primer F, Sangon Biotech) and calculated using the 2^−ΔΔCt^ method. The primer sequences are shown in Supplementary Table S1.

### 2.8 Double luciferase reporter gene assay

The wild type (WT) and mutant (MU) of the target 3′-UTR were cloned and inserted into the pSI-Check2 vector (Promega), and the successful construction of the plasmid was verified by sequencing. Before transfection, 293T cells and the target plasmid were seeded in a 96-well plate. Then, LipoFiter 3.0 (Hanbio) was used to cotransfect the WT and MU plasmids with the miR-1-3p plasmid. The dual luciferase reporter gene assay system (Promega) was used to evaluate the activities of firefly luciferase and Renilla luciferase.

### 2.9 Statistical analysis

Normally distributed data are expressed as the mean ± standard deviation (mean ± SD); nonnormally distributed data are expressed as the median (lower quartile, upper quartile). The comparison between the two groups was performed by *t* test or Mann–Whitney test. *p* < 0.05 was regarded as significantly different.

## 3 Results

### 3.1 Ventricular septal defect group microRNAs expression profile and bioinformatics analysis

Of the myocardium and amniotic fluid samples (divided into NC group, non-VSD group, and VSD group) and maternal serum samples (divided into NC group and VSD group), seven samples were selected for miRNA sequencing in each group.

#### 3.1.1 MicroRNAs expression profile

**FIGURE 2:**
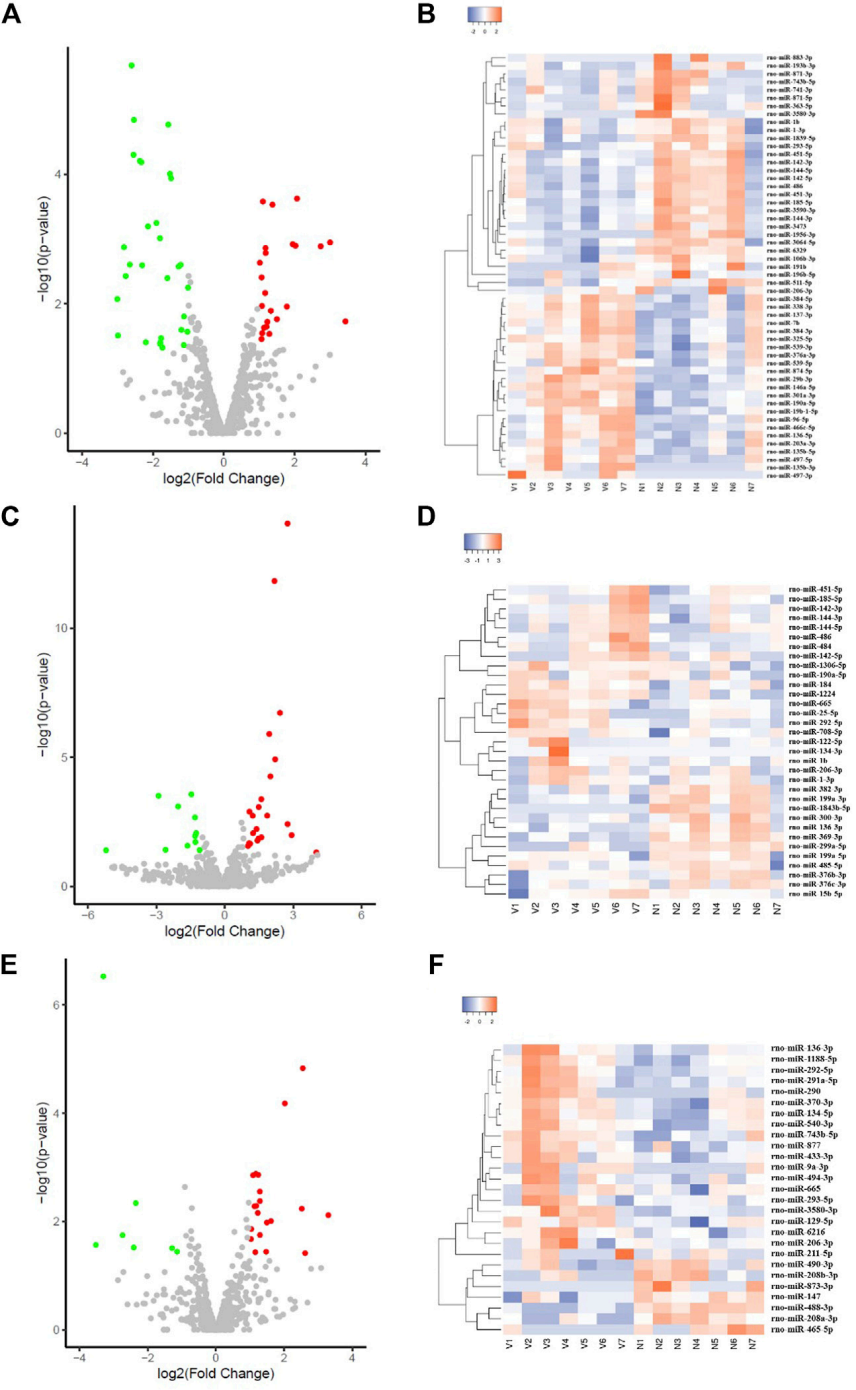
Volcano plot and cluster heatmap of DE-microRNAs in the VSD group **(A,C,E)** are all volcano plots; **(B,D,F)** are cluster heatmaps; **(A,B)** represents myocardium-derived DE-microRNAs; **(C,D)** represents amniotic fluid-derived DE-microRNAs; **(E,F)** represents maternal serum-derived DE-microRNAs.

Compared with the NC group, there were 53 myocardium-derived DE-microRNAs in the VSD group, of which 23 were upregulated (miR-497-3p, miR-7b, etc.) and 30 were downregulated (miR-1-3p, miR-1b, miR-293-5p and miR-3580-3p, etc., Supplementary Table S2; Figures 2A,B). There were 34 DE-microRNAs in the non-VSD group, including 9 upregulated and 25 downregulated microRNAs (Supplementary Table S3).

There were 33 amniotic fluid-derived DE-microRNAs in the VSD group, including 22 upregulated (miR-15b-5p, miR-1b, etc.) and 11 downregulated microRNAs (miR-1843b-5p, miR-299a-5p, etc., Supplementary Table S4; Figures 2C,D). There were 48 DE-microRNAs in the non-VSD group, including 39 upregulated and 9 downregulated microRNAs (Supplementary Table S5).

Twenty-seven maternal serum-derived DE-microRNAs were detected in the VSD group, including 20 upregulated (miR-129-5p, miR-206-3p, miR-293-5p, miR-3580-3p, miR-494-3p, etc.), and 7 downregulated microRNAs (miR-208a-3p, miR-208b-3p, etc., Supplementary Table S6; Figures 2E,F).

#### 3.1.2 Target gene prediction and bioinformatics analysis of differentially expressed microRNAs from different samples

Considering the possible effects of DMO on microRNA expression in the fetus, DE-microRNAs in the myocardium and amniotic fluid of the non-VSD group were excluded from the target gene prediction and bioinformatics analysis, and the unique DE-microRNAs in the VSD group were analyzed.

There were 35 unique DE-microRNAs in the myocardium of the VSD group, of which 20 were upregulated and 15 were downregulated. A total of 10,429 target genes of these DE-microRNAs were identified by miRWalk3.0, of which 207 related to VSD were recorded in disease-related databases (including OMIM, KEGG Disease database and GWAS Catalog database). Twenty unique DE-microRNAs of the VSD group were discovered in amniotic fluid, 10 of which were upregulated and 10 of which were downregulated. A total of 9,352 target genes were predicted, of which 179 were related to VSD. Maternal serum-derived DE-microRNAs predicted a total of 11,317 target genes, of which 231 were related to VSD.

GO analysis of the abovementioned VSD-related target genes showed that the target genes of different samples were significantly enriched in biological process (BP), such as heart development, cardiac chamber development, cardiac chamber morphogenesis, and circulatory system development. Cellular component (CC) analysis showed that they are mainly located in the membrane-bounded organelle, nucleoplasm and nucleus and perform molecular function (MF), such as chromatin binding, transcription factor binding and protein binding. KEGG analysis indicated that in addition to participating in cancer-related pathways, the abovementioned genes were also enriched in pathways that regulate pluripotency of stem cells, Ras related to heart disease, and MAPK related to cardiomyocyte proliferation (Supplementary Figure S1).

#### 3.1.3 Construction and analysis of protein–protein interaction network

The PPI network of the protein expression of target genes was constructed. Combining the 7 algorithms of cytoHubba (including MCC, DMNC, MNC, Degree, EPC, Closeness and Radiality), the top 20 hub genes in the PPI network were screened out, and the intersection was assessed. The hub genes in the myocardium are *Kras*, *Map2k1*, *Fgfr1*, *Ptpn11* and *Igf1r*, and the corresponding myocardium-derived DE-microRNAs are miR-3580-3p, miR-497-3p and miR-96-5p, etc. The hub genes in amniotic fluid are *Map2k1*, *Abl1*, *Cxcr4* and *Tek*, corresponding to the amniotic fluid-derived DE-microRNAs are miR-199a-5p and miR-184, etc. The hub genes in maternal serum are *Ptpn11*, *Fgfr1*, *Igf1r* and *Map2k1*, and the corresponding maternal serum-derived DE-microRNAs are miR-3580-3p and miR-494-3p, etc. (Supplementary Table S7; Table 2).

**TABLE 2:** DE-microRNAs related to hub genes.

	Related DE-microRNAs
Myocardium	miR-142-3p, miR-1839-5p, miR-185-5p, miR-301a-3p, miR-3064-5p, miR-325-5p, miR-3473, miR-3580-3p, miR-363-5p, miR-384-5p, miR-497-3p, miR-741-3p, miR-874-5p, miR-96-5p
Amniotic fluid	miR-122-5p, miR-134-3p, miR-184, miR-1843b-5p, miR-199a-5p, miR-299a-5p, miR-665
Serum	miR-3580-3p, miR-370-3p, miR-433-3p, miR-494-3p, miR-6216, miR-665, miR-873-3p, miR-877

**FIGURE 3:**
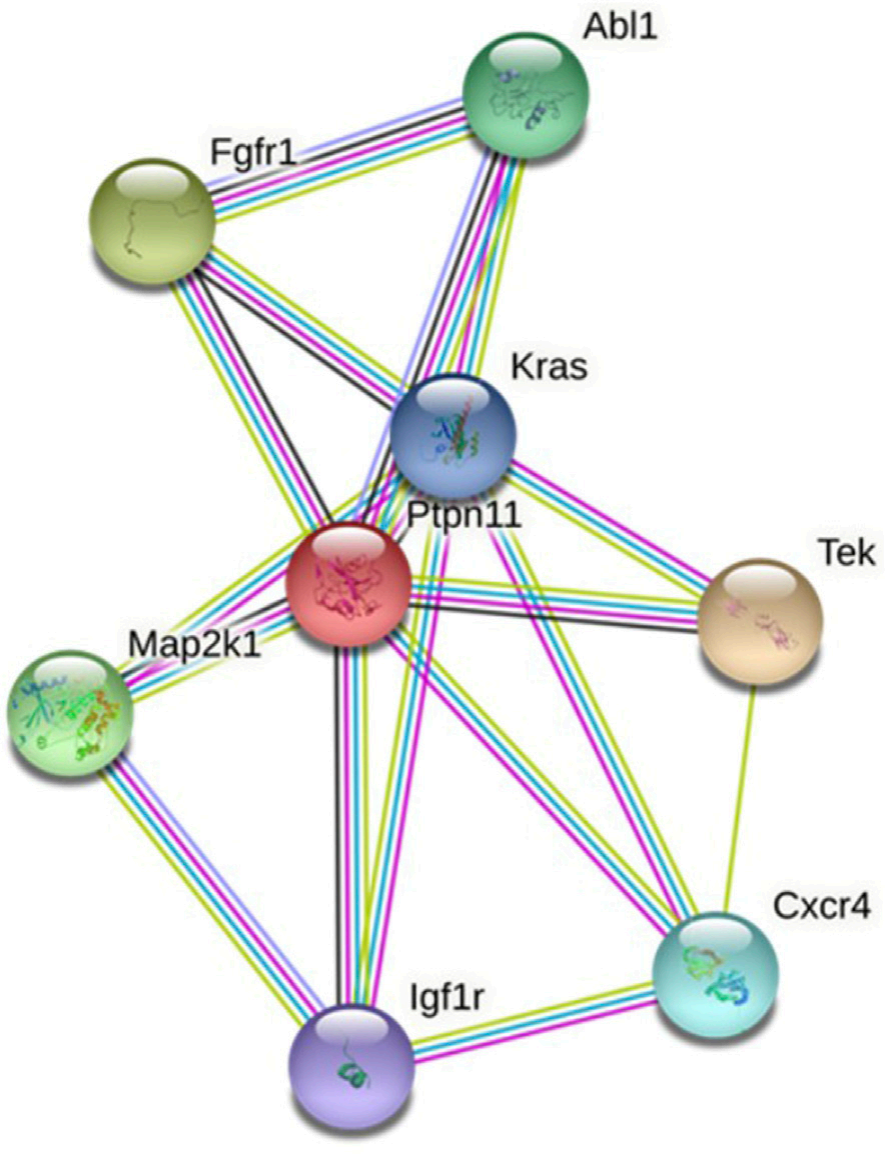
PPI network of proteins coded by key target genes.

Hub genes were synthesized in three kinds of samples to construct a PPI network, elucidating the relationship and interaction among these proteins (Figure 3).

#### 3.1.4 Quantitative real-time polymerase chain reaction confirmed the expression of differentially expressed microRNAs

**FIGURE 4:**
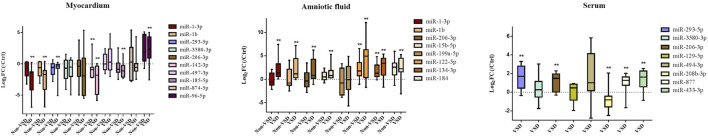
qRT–PCR verified the expression of DE-microRNAs. (Compared with NC group: *p < 0.05, **p < 0.01. *n* = 9)

The expression of DE-microRNAs, which were differentially expressed in both the myocardium and circulation, or were associated with VSD according to previous studies, or related to hub genes, were verified in myocardium, amniotic fluid and maternal serum (Figure 4). Each group consisted of nine samples. MiR-1-3p, miR-1b, and miR-293-5p are DE-microRNAs differentially expressed in both the myocardium and amniotic fluid/maternal serum. Among them, miR-1-3p and miR-1b are downregulated in the myocardium but upregulated in the amniotic fluid. The expression of miR-293-5p is downregulated in myocardium but upregulated in maternal serum. MiR-3580-3p has a similar expression trend as miR-293-5p in the myocardium and serum, and the difference was not significant. In addition, miR-206-3p was significantly overexpressed in amniotic fluid and maternal serum. MiR-185-5p and miR-96-5p were differentially expressed in myocardium. MiR-15b-5p and miR-184 were differentially expressed in amniotic fluid. MiR-208b-3p, miR-877 and miR-433-3p were differentially expressed in maternal serum. MiR-142-3p, miR-122-5p and miR-134-3p showed differential expression in both VSD group and non-VSD group.

### 3.2 Expression of amniotic fluid-derived differentially expressed microRNAs in clinical samples

Amniotic fluid-derived DE-microRNAs that were verified by quantitative real-time polymerase chain reaction (qRT–PCR) were selected. BLAST (v2.8.1) was used to identify homologous human miRNAs and confirm conservation (Supplementary Table S8).

**FIGURE 5:**
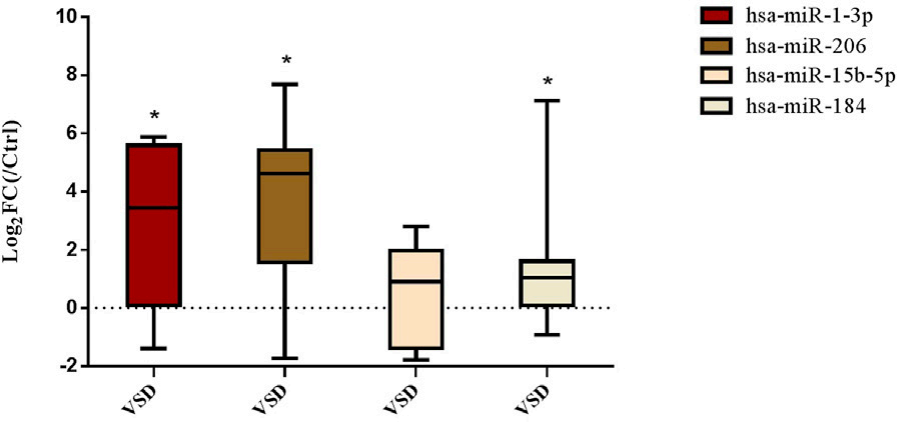
Expression of amniotic fluid-derived DE-microRNAs in clinical samples. (Compared with NC group: *p < 0.05. *n* = 7)

Seven human fetuses were diagnosed with perimembranous VSD or muscular VSD by prenatal ultrasound examination, and the diagnosis was confirmed by follow-up after birth. The size of defect ranged from 1.2 to 4.8 mm. It was confirmed in clinical amniotic fluid samples that miR-1-3p, miR-206 and miR-184 were also overexpressed in clinical amniotic fluid samples (Figure 5).

### 3.3 *SLC8A1* is the target gene of miR-1-3p

**FIGURE 6:**
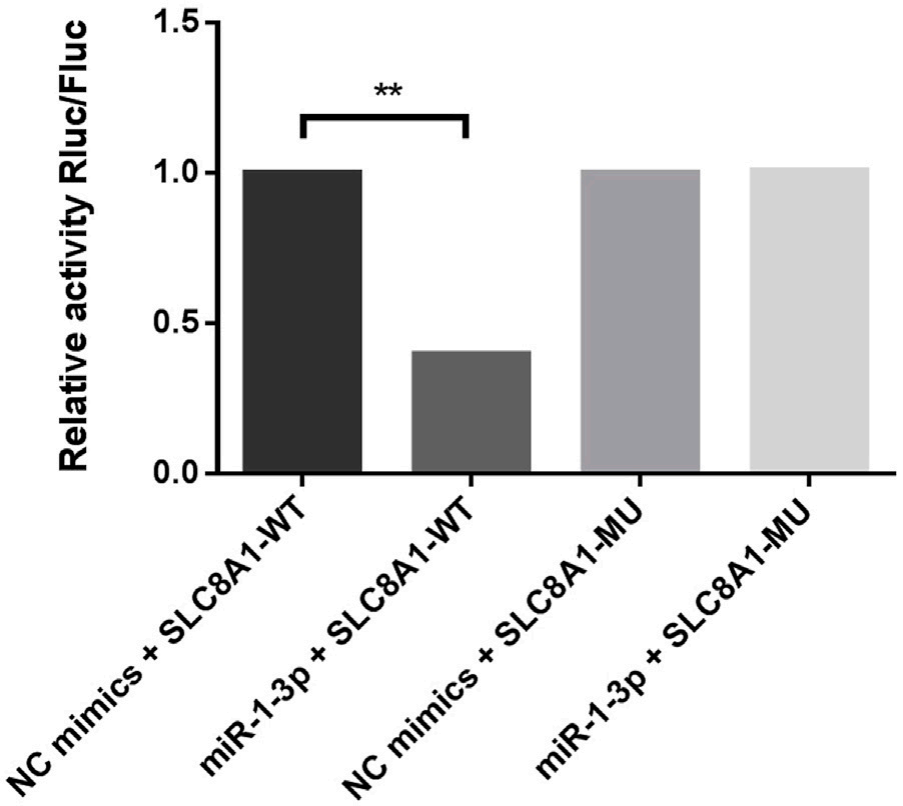
Dual luciferase reporter assay verifies that SLC8A1 is the direct target of miR-1-3p. (MiR-1-3p significantly reduced the luciferase activity of plasmids containing the WT SLC8A1 3′UTR. Fluc, firefly luciferase activity; Rluc, Renilla luciferase activity; WT, wild type; MU, mutant. **p < 0.01)

MiR-1-3p, which is differentially expressed in both the myocardium and amniotic fluid, was selected for target gene prediction. Solute carrier family 8 member A1 (*SLC8A1*, also known as sodium-calcium exchanger, *NCX1*) may be the target gene of miR-1-3p. The 3′-UTR of *SLC8A1* was cloned and inserted into the pSI-Check2 vector to construct a recombinant plasmid. The dual luciferase reporter system was used to detect the relative luciferase activity. It was demonstrated that miR-1-3p can significantly reduce the luciferase activity of the plasmid containing the wild-type *SLC8A1* 3′UTR but has no significant effect on the plasmid containing the mutant *SLC8A1* 3′UTR, indicating that miR-1-3p can inhibit luciferase activity by binding to the 3′UTR of *SLC8A1*. Consequently, *SLC8A1* is the direct target of miR-1-3p (Figure 6).

## 4 Discussion

In this study, we detected and analyzed the expression profiles of miRNAs in the myocardium, amniotic fluid and maternal serum of VSD fetal rats and found that miR-1-3p, miR-1b and miR-293-5p were differentially expressed in amniotic fluid/maternal serum and the myocardium. The expression of amniotic fluid-derived DE-microRNAs (miR-1-3p, miR-206 and miR-184) was confirmed in clinical samples. Then, it was predicted and verified that one of the target genes of miR-1-3p is *SLC8A1/NCX1*, which is related to cardiomyocyte apoptosis, indicating that circulating miRNAs involved in the regulation of VSD may be derived from the myocardium, providing a theoretical basis for the use of circulating miRNAs to assist in the diagnosis of VSD.

MiRNAs participate in heart development, and their dysregulation may be related to CHD (Bruneau, 2008). The absence of the miRNA processing enzyme Dicer can lead to abnormal formation of the heart outflow tract and chambers in mammals, so miRNA is essential for heart development (Saxena and Tabin, 2010). Previous studies have revealed that a variety of miRNAs are involved in processes such as heart development and cardiomyocyte proliferation and differentiation, which are closely related to CHDs, including VSD (Wang et al., 2016; Toni et al., 2020; Zhuang et al., 2020).

MiR-1 is a myocardial and skeletal muscle-specific miRNA (Townley-Tilson et al., 2010). Li et al. (2013) analyzed the expression of miR-1-1 in the human heart and found that the overexpression of *GJA1* and *SOX9* was correlated with the decrease in miR-1-1 in VSD, indicating that miR-1-1 regulates the above target genes related to the pathogenesis of VSD. In patients with tetralogy of Fallot (TOF), miR-1 and miR-133 are significantly downregulated, which is predicted to affect the development and function of the heart by regulating genes such as *KCNJ2*, *FBN2*, *SLC38A3* and *TNNI1* (Grunert et al., 2019). Therefore, the downregulation of miR-1 expression is closely associated with heart development. In contrast, it was reported that the overexpression of miR-1 and miR-133 effectively promotes the reprogramming of fibroblasts to cardiomyocytes, reduces apoptosis and increases the viability of P19 cells differentiated into cardiomyocytes (Liu et al., 2017; Riching et al., 2021). In a study on circulating miRNA, Stoica et al. (2020) detected plasma miR-1 in children with CHDs who underwent surgery and found that high expression of plasma miR-1 was associated with longer intensive care time, more serious cardiovascular events and an increasing ventilation index. Hence, miR-1 can be an indicator to evaluate the prognosis of children after surgery.

Our results supported the above reports. In the myocardium of VSD fetal rats, the expression of miR-1-3p and miR-1b, both of which belong to the mir-1 family, was significantly downregulated but was upregulated in the amniotic fluid of rats and humans. This finding of circulating miR-1 is similar to the findings of the study of Stoica et al. (2020). Furthermore, a dual luciferase reporter system was used to verify that miR-1-3p participates in the regulation of VSD by directly targeting *SLC8A1/NCX1*. *SLC8A1/NCX1* is highly expressed in the myocardium and is responsible for expelling Ca^2+^ from cardiomyocytes during diastole, which is the main mechanism by which cardiomyocytes return to a resting state after excitation (Wakimoto et al., 2000; Fagerberg et al., 2014). Studies have shown that *SLC8A1/NCX1* deficiency results in death and cardiomyocyte apoptosis in mice in the middle embryonic stage, suggesting that *SLC8A1/NCX1* is essential for fetuses and embryonic cardiomyocyte survival (Wakimoto et al., 2000). In addition, *SLC8A1/NCX1* can induce a variety of heart defects, such as VSD and arrhythmia (Raveau et al., 2012). It was reported that *SLC8A1/NCX1* elevated in ductus arteriosus (DA)-dependent CHDs, and play a role in preventing DA functional closure and delaying the anatomical closure process (Li et al., 2017). Previous studies showed that overexpression of *SLC8A1/NCX1* was associated with myocardial hypertrophy (Tyser et al., 2016; Ottolia et al., 2021). Roos et al. (2007) indicated that increased expression of the *SLC8A1/NCX1* directly led to heart hypertrophy, and the magnitude of the hypertrophy and pathology increased with increasing *SLC8A1/NCX1* expression. It is almost always accompanied by loss of myocytes (apoptosis and/or necrosis) in hypertrophy progresses (Roos et al., 2007). He et al. (2015) also proved that *SLC8A1/NCX1* in mouse with myocardial hypertrophy is increased and positively correlated with the cardiomyocyte apoptosis. Therefore, the overexpression of *SLC8A1/NCX1* could lead to hypertrophy, apoptosis and/or necrosis. Our study further confirmed that miR-1-3p directly targets *SLC8A1/NCX1* in the myocardium and participates in the regulation of cardiac electrophysiological activities and cardiomyocyte apoptosis, which is closely related to the pathogenesis of VSD.

This study also verified the upregulation of miR-293-5p, miR-15b-5p, miR-206, miR-184, miR-877 and miR-433-3p and the downregulation of miR-208b-3p in circulation. The miRNAs mentioned above have been shown to regulate the pluripotency of stem cells, the differentiation and apoptosis of cardiomyocytes and other processes related to the occurrence of heart diseases. MiR-293-5p was differentially expressed in both the myocardium and maternal serum. MiR-293-5p is a member of the miR-290–295 cluster that is most abundantly expressed in rat pluripotent stem cells and is involved in the regulation of pluripotency and reprogramming in rats (Sherstyuk et al., 2017). MiR-15b may be upregulated due to apoptosis induced by cardiac ischaemia/reperfusion injury (Liu et al., 2012; Liu et al., 2014). Zhao et al. (2019) found that miR-15b can directly target the 3′-UTR of *SETD3* to inhibit its expression and myoblast differentiation, while downregulation of miR-15b can promote myoblast differentiation. MiR-208b is mainly expressed in the embryonic heart and skeletal muscle. The expression of miR-208b decreases during embryonic development to adulthood, while an increase in the adult heart may be related to pathological remodelling of the heart in dilated cardiomyopathy (Zhou et al., 2017). MiR-208b-3p participated in miR-208b-3p/Med13/Wnt/β-catenin signaling pathway axis and against hypoxia/reoxygenation injury. MiR-206 is significantly decreased in the peripheral blood of TOF patients after surgery (Abu-Halima et al., 2017). MiR-184 was increased in cardiomyocytes suffering from oxidative stress, and inhibition of miR-184 could inhibit cardiomyocyte apoptosis (Zou et al., 2020a). MiR-877 were differentially expressed in the right ventricle of pulmonary arterial hypertension rats (Joshi et al., 2016). MiR-433-3p upregulated in patients with critical coronary stenosis (Infante et al., 2019). Accordingly, our study indicated that DE-microRNAs (miR-293-5p, miR-15b-5p, miR-206, miR-208b-3p, etc.) in the circulation of amniotic fluid and maternal serum are related to VSD, which is helpful for the prenatal diagnosis of fetal VSD.

Scholars have proposed that circulating miRNAs can be packaged in microvesicles, exosomes, and apoptotic bodies or combined with RNA-binding proteins or lipoprotein complexes to maintain stability and prevent degradation (Creemers et al., 2012; Tsatsaronis et al., 2018; Mori et al., 2019). Previous studies of arrhythmogenic cardiomyopathy, acute myocardial infarction, liver injury, tumours and other diseases have found that miRNAs are underexpressed in tissue but overexpressed in circulation, indicating that circulating DE-microRNAs may originate from damaged tissues or apoptotic cells (Bueno Marinas et al., 2020; Zou et al., 2020b). Apoptosis play an important role in the pathogenesis of VSD. The increase of apoptosis would result in cardiac defects, including VSD (Chen et al., 2009). Studies revealed that increased apoptosis observed in interventricular septum or outflow tract cushions was likely contributing to VSD (Gaussin et al., 2002; Liang et al., 2007). It was reported that apoptotic cells were increased in VSD models. Kumar et al. (2007) found that the apoptotic cells were significantly increased in the ventricular myocardium of the streptozotocin-induced diabetic mice embryos. The increased apoptosis was also confirmed in several genetically mutated models of VSDs (Chen et al., 2009; Huang et al., 2012; Liu et al., 2018). In this study, miR-1-3p, miR-1b and miR-293-5p all showed similar low expression in the myocardium of VSD fetal mice and high expression in amniotic fluid and maternal serum, which supports this hypothesis and indicates that the miRNAs mentioned above may be actively or passively released into the circulation by cardiomyocytes, suggesting myocardial injury or apoptosis. Therefore, the overexpression of miRNAs such as miR-1-3p, miR-1b and miR-293-5p in the circulation is a direct and powerful clue related to fetal VSD.

This study has certain limitations. Some miRNAs considered to be related to VSD in previous studies (such as miR-133, miR-181c, etc.) were not greatly differentially expressed in this study. The diversity may be because the samples in this study were derived from fetal mice and related body fluids, while some of the previous studies selected different samples, such as VSD patients after birth and cells cultured *in vitro*, or different pretreatment methods, such as whole blood and exosomes of amniotic fluid/serum. This study verified that one of the target genes of miR-1-3p is *SLC8A1/NCX1* by the dual luciferase reporter system. The expression and regulatory pathways of miR-1-3p and *SLC8A1/NCX1* in VSD fetus remains to be further explored.

## 5 Conclusion

Our study comprehensively analyzed the expression profile of miRNAs in the myocardium, amniotic fluid and maternal serum and found that miR-1-3p, miR-1b and miR-293-5p were differentially expressed in both the myocardium and circulation and may be released by necrotic or apoptotic cardiomyocytes and thus appear to be downregulated in the myocardium and upregulated in the circulation. MiR-1-3p targeting *SLC8A1* participates in the regulation of VSD, and the same expression was confirmed in human amniotic fluid, which indicates that miRNA has great potential to become a biomarker for the prenatal diagnosis of VSD. This finding provided a foundation and broadened the horizon of the use of circulating miRNAs to assist in the prenatal diagnosis of VSD. The value in clinical application is expected to be verified in long-term follow-up of new cohorts in the future.

## Data Availability

The datasets presented in this study can be found in online repositories. The names of the repository/repositories and accession number(s) can be found below: NCBI GEO - GSE194240.

## References

[B1] Abu-Halima M. Meese E. Keller A. Abdul-Khaliq H. Rädle-Hurst T. (2017). Analysis of circulating microRNAs in patients with repaired Tetralogy of Fallot with and without heart failure. J. Transl. Med. 15, 156. 10.1186/s12967-017-1255-z 28693530 PMC5504636

[B2] Bruneau B. G. (2008). The developmental genetics of congenital heart disease. Nature 451, 943–948. 10.1038/nature06801 18288184

[B3] Bueno Marinas M. Celeghin R. Cason M. Bariani R. Frigo A. C. Jager J. (2020). A microRNA expression profile as non-invasive biomarker in a large arrhythmogenic cardiomyopathy cohort. Int. J. Mol. Sci. 21, E1536. 10.3390/ijms21041536 32102357 PMC7073183

[B4] Chen Q. Chen H. Zheng D. Kuang C. Fang H. Zou B. (2009). Smad7 is required for the development and function of the heart. J. Biol. Chem. 284, 292–300. 10.1074/jbc.M807233200 18952608 PMC2610499

[B5] Cox K. Algaze-Yojay C. Punn R. Silverman N. (2020). The natural and unnatural history of ventricular septal defects presenting in infancy: An echocardiography-based review. J. Am. Soc. Echocardiogr. 33, 763–770. 10.1016/j.echo.2020.01.013 32249125

[B6] Creemers E. E. Tijsen A. J. Pinto Y. M. (2012). Circulating microRNAs: Novel biomarkers and extracellular communicators in cardiovascular disease? Circ. Res. 110, 483–495. 10.1161/CIRCRESAHA.111.247452 22302755

[B7] Fagerberg L. Hallström B. M. Oksvold P. Kampf C. Djureinovic D. Odeberg J. (2014). Analysis of the human tissue-specific expression by genome-wide integration of transcriptomics and antibody-based proteomics. Mol. Cell. Proteomics 13, 397–406. 10.1074/mcp.M113.035600 24309898 PMC3916642

[B8] Gaussin V. Van de Putte T. Mishina Y. Hanks M. C. Zwijsen A. Huylebroeck D. (2002). Endocardial cushion and myocardial defects after cardiac myocyte-specific conditional deletion of the bone morphogenetic protein receptor ALK3. Proc. Natl. Acad. Sci. U. S. A. 99, 2878–2883. 10.1073/pnas.042390499 11854453 PMC122441

[B9] Grunert M. Appelt S. Dunkel I. Berger F. Sperling S. R. (2019). Altered microRNA and target gene expression related to Tetralogy of Fallot. Sci. Rep. 9, 19063. 10.1038/s41598-019-55570-4 31836860 PMC6911057

[B10] Grunert M. Dorn C. Cui H. Dunkel I. Schulz K. Schoenhals S. (2016). Comparative DNA methylation and gene expression analysis identifies novel genes for structural congenital heart diseases. Cardiovasc. Res. 112, 464–477. 10.1093/cvr/cvw195 27496870

[B11] He J. Cai Y. Luo L. M. Wang R. (2015). Expression of Wnt and NCX1 and its correlation with cardiomyocyte apoptosis in mouse with myocardial hypertrophy. Asian pac. J. Trop. Med. 8, 930–936. 10.1016/j.apjtm.2015.10.002 26614993

[B12] Huang X. Huang F. Yang D. Dong F. Shi X. Wang H. (2012). Expression of microRNA-122 contributes to apoptosis in H9C2 myocytes. J. Cell. Mol. Med. 16, 2637–2646. 10.1111/j.1582-4934.2012.01577.x 22453009 PMC4118232

[B13] Hui L. Slonim D. K. Wick H. C. Johnson K. L. Bianchi D. W. (2012). The amniotic fluid transcriptome: A source of novel information about human fetal development. Obstet. Gynecol. 119, 111–118. 10.1097/AOG.0b013e31823d4150 22183218 PMC3273331

[B14] Infante T. Forte E. Punzo B. Cademartiri F. Cavaliere C. Soricelli A. (2019). Correlation of circulating miR-765, miR-93-5p, and miR-433-3p to obstructive coronary heart disease evaluated by cardiac computed tomography. Am. J. Cardiol. 124, 176–182. 10.1016/j.amjcard.2019.04.016 31084998

[B15] Islas J. F. Moreno-Cuevas J. E. (2018). A MicroRNA perspective on cardiovascular development and diseases: An update. Int. J. Mol. Sci. 19, 2075. 10.3390/ijms19072075 PMC6073753 30018214

[B16] Jin Y. Ai L. Chai X. Tang P. Zhang W. Yang L. (2021). Maternal circulating exosomal miRNAs as non-invasive biomarkers for the prediction of fetal ventricular septal defect. Front. Genet. 12, 717208. 10.3389/fgene.2021.717208 34567071 PMC8458870

[B17] Joshi S. R. Dhagia V. Gairhe S. Edwards J. G. McMurtry I. F. Gupte S. A. (2016). MicroRNA-140 is elevated and mitofusin-1 is downregulated in the right ventricle of the Sugen5416/hypoxia/normoxia model of pulmonary arterial hypertension. Am. J. Physiol. Heart Circ. Physiol. 311, H689–H698. 10.1152/ajpheart.00264.2016 27422986 PMC7199238

[B18] Kumar S. D. Dheen S. T. Tay S. S. (2007). Maternal diabetes induces congenital heart defects in mice by altering the expression of genes involved in cardiovascular development. Cardiovasc. Diabetol. 6, 34. 10.1186/1475-2840-6-34 17967198 PMC2176054

[B19] Li J. Cao Y. Ma X. J. Wang H. J. Zhang J. Luo X. (2013). Roles of miR-1-1 and miR-181c in ventricular septal defects. Int. J. Cardiol. 168, 1441–1446. 10.1016/j.ijcard.2012.12.048 23352489

[B20] Li M. Jiang C. Ye L. Wang S. Zhang H. Liu J. (2017). The role of Na+/Ca2+ exchanger 1 in maintaining ductus arteriosus patency. Sci. Rep. 7, 9826. 10.1038/s41598-017-10377-z 28852106 PMC5575298

[B21] Liang X. Sun Y. Schneider J. Ding J. H. Cheng H. Ye M. (2007). Pinch1 is required for normal development of cranial and cardiac neural crest-derived structures. Circ. Res. 100, 527–535. 10.1161/01.RES.0000259041.37059.8c 17272814 PMC5837278

[B22] Liu F. Liu X. Xu Z. Yuan P. Zhou Q. Jin J. (2018). Molecular mechanisms of Ellis-van Creveld gene variations in ventricular septal defect. Mol. Med. Rep. 17 (1), 1527–1536. 10.3892/mmr.2017.8088 29257216 PMC5780092

[B23] Liu L. F. Liang Z. Lv Z. R. Liu X. H. Bai J. Chen J. (2012). MicroRNA-15a/b are up-regulated in response to myocardial ischemia/reperfusion injury. J. Geriatr. Cardiol. 9, 28–32. 10.3724/SP.J.1263.2012.00028 22783320 PMC3390100

[B24] Liu L. Yuan Y. He X. Xia X. Mo X. (2017). MicroRNA-1 upregulation promotes myocardiocyte proliferation and suppresses apoptosis during heart development. Mol. Med. Rep. 15, 2837–2842. 10.3892/mmr.2017.6282 28260051

[B25] Liu L. Zhang G. Liang Z. Liu X. Li T. Fan J. (2014). MicroRNA-15b enhances hypoxia/reoxygenation-induced apoptosis of cardiomyocytes via a mitochondrial apoptotic pathway. Apoptosis 19, 19–29. 10.1007/s10495-013-0899-2 24043355

[B26] Meng X. Zhang P. Zhang L. (2020). Fetal hypoxia impacts on proliferation and differentiation of sca-1(+) cardiac progenitor cells and maturation of cardiomyocytes: A role of MicroRNA-210. Genes 11, 328. 10.3390/genes11030328 PMC7140790 32244901

[B27] Mori M. A. Ludwig R. G. Garcia-Martin R. Brandão B. B. Kahn C. R. (2019). Extracellular miRNAs: From biomarkers to mediators of physiology and disease. Cell Metab. 30, 656–673. 10.1016/j.cmet.2019.07.011 31447320 PMC6774861

[B28] Ottolia M. John S. Hazan A. Goldhaber J. I. (2021). The cardiac Na(+) -Ca(2+) exchanger: From structure to function. Compr. Physiol. 12, 2681–2717. 10.1002/cphy.c200031 34964124 PMC8773166

[B29] Panni S. Lovering R. C. Porras P. Orchard S. (2020). Non-coding RNA regulatory networks. Biochim. Biophys. Acta. Gene Regul. Mech. 1863, 194417. 10.1016/j.bbagrm.2019.194417 31493559

[B30] Pulignani S. Andreassi M. G. (2019). MicroRNAs and congenital heart disease: Where are we now? Rev. Esp. Cardiol. 72, 7–9. 10.1016/j.rec.2018.06.030 30056121

[B31] Raveau M. Lignon J. M. Nalesso V. Duchon A. Groner Y. Sharp A. J. (2012). The App-Runx1 region is critical for birth defects and electrocardiographic dysfunctions observed in a Down syndrome mouse model. PLoS Genet. 8, e1002724. 10.1371/journal.pgen.1002724 22693452 PMC3364940

[B32] Riching A. S. Danis E. Zhao Y. Cao Y. Chi C. Bagchi R. A. (2021). Suppression of canonical TGF-β signaling enables GATA4 to interact with H3K27me3 demethylase JMJD3 to promote cardiomyogenesis. J. Mol. Cell. Cardiol. 153, 44–59. 10.1016/j.yjmcc.2020.12.005 33359755 PMC8809092

[B33] Roos K. P. Jordan M. C. Fishbein M. C. Ritter M. R. Friedlander M. Chang H. C. (2007). Hypertrophy and heart failure in mice overexpressing the cardiac sodium-calcium exchanger. J. Card. Fail. 13, 318–329. 10.1016/j.cardfail.2007.01.004 17517353 PMC2017112

[B34] Sabour D. Machado R. S. R. Pinto J. P. Rohani S. Sahito R. G. A. Hescheler J. (2018). Parallel genome-wide profiling of coding and non-coding RNAs to identify novel regulatory elements in embryonic and maturated heart. Mol. Ther. Nucleic Acids 12, 158–173. 10.1016/j.omtn.2018.04.018 30195755 PMC6023836

[B35] Saxena A. Tabin C. J. (2010). miRNA-processing enzyme Dicer is necessary for cardiac outflow tract alignment and chamber septation. Proc. Natl. Acad. Sci. U. S. A. 107, 87–91. 10.1073/pnas.0912870107 20018673 PMC2806718

[B36] Sherstyuk V. V. Medvedev S. P. Elisaphenko E. A. Vaskova E. A. Ri M. T. Vyatkin Y. V. (2017). Genome-wide profiling and differential expression of microRNA in rat pluripotent stem cells. Sci. Rep. 7, 2787. 10.1038/s41598-017-02632-0 28584262 PMC5459850

[B37] Smith T. Rajakaruna C. Caputo M. Emanueli C. (2015). MicroRNAs in congenital heart disease. Ann. Transl. Med. 3, 333. 10.3978/j.issn.2305-5839.2015.12.25 26734643 PMC4690991

[B38] Song Y. Higgins H. Guo J. Harrison K. Schultz E. N. Hales B. J. (2018). Clinical significance of circulating microRNAs as markers in detecting and predicting congenital heart defects in children. J. Transl. Med. 16, 42. 10.1186/s12967-018-1411-0 29482591 PMC5828434

[B39] Spicer D. E. Hsu H. H. Co-Vu J. Anderson R. H. Fricker F. J. (2014). Ventricular septal defect. Orphanet J. Rare Dis. 9, 144. 10.1186/s13023-014-0144-2 25523232 PMC4316658

[B40] Stoica S. C. Dorobantu D. M. Vardeu A. Biglino G. Ford K. L. Bruno D. V. (2020). MicroRNAs as potential biomarkers in congenital heart surgery. J. Thorac. Cardiovasc. Surg. 159, 1532–1540. 10.1016/j.jtcvs.2019.03.062 31043318

[B41] Thomford N. E. Dzobo K. Yao N. A. Chimusa E. Evans J. Okai E. (2018). Genomics and Epigenomics of congenital heart defects: Expert review and lessons learned in africa. OMICS 22, 301–321. 10.1089/omi.2018.0033 29762087 PMC6016577

[B42] Toni L. S. Hailu F. Sucharov C. C. (2020). Dysregulated micro-RNAs and long noncoding RNAs in cardiac development and pediatric heart failure. Am. J. Physiol. Heart Circ. Physiol. 318, H1308–H1315. 10.1152/ajpheart.00511.2019 32216613 PMC7346540

[B43] Townley-Tilson W. H. Callis T. E. Wang D. (2010). MicroRNAs 1, 133, and 206: Critical factors of skeletal and cardiac muscle development, function, and disease. Int. J. Biochem. Cell Biol. 42, 1252–1255. 10.1016/j.biocel.2009.03.002 20619221 PMC2904322

[B44] Tsatsaronis J. A. Franch-Arroyo S. Resch U. Charpentier E. (2018). Extracellular vesicle RNA: A universal mediator of microbial communication? Trends Microbiol. 26, 401–410. 10.1016/j.tim.2018.02.009 29548832

[B45] Tyser R. C. Miranda A. M. Chen C. M. Davidson S. M. Srinivas S. Riley P. R. (2016). Calcium handling precedes cardiac differentiation to initiate the first heartbeat. eLife 5, e17113. 10.7554/eLife.17113 27725084 PMC5059139

[B46] van Nisselrooij A. E. L. Teunissen A. K. K. Clur S. A. Rozendaal L. Pajkrt E. Linskens I. H. (2020). Why are congenital heart defects being missed? Ultrasound Obstet. Gynecol. 55, 747–757. 10.1002/uog.20358 31131945 PMC7317409

[B47] Wakimoto K. Kobayashi K. Kuro O. M. Yao A. Iwamoto T. Yanaka N. (2000). Targeted disruption of Na+/Ca2+ exchanger gene leads to cardiomyocyte apoptosis and defects in heartbeat. J. Biol. Chem. 275, 36991–36998. 10.1074/jbc.M004035200 10967099

[B48] Wang L. Song G. Liu M. Chen B. Chen Y. Shen Y. (2016). MicroRNA-375 overexpression influences P19 cell proliferation, apoptosis and differentiation through the Notch signaling pathway. Int. J. Mol. Med. 37, 47–55. 10.3892/ijmm.2015.2399 26531318 PMC4687438

[B49] Wang Z. Yang Y. Xiong W. Zhou R. Song N. Liu L. (2020). Dexmedetomidine protects H9C2 against hypoxia/reoxygenation injury through miR-208b-3p/Med13/Wnt signaling pathway axis. Biomed. Pharmacother. = Biomedecine Pharmacother. 125, 110001. 10.1016/j.biopha.2020.110001 32070878

[B50] Yang H. Yang S. Shen H. Wu S. Ruan J. Lyu G. (2021). Construction of the amniotic fluid-derived exosomal ceRNA network associated with ventricular septal defect. Genomics 113, 4293–4302. 10.1016/j.ygeno.2021.11.003 34758360

[B51] Zhao M. J. Xie J. Shu W. J. Wang H. Y. Bi J. Jiang W. (2019). MiR-15b and miR-322 inhibit SETD3 expression to repress muscle cell differentiation. Cell Death Dis. 10, 183. 10.1038/s41419-019-1432-5 30796205 PMC6385263

[B52] Zhou Q. Schötterl S. Backes D. Brunner E. Hahn J. K. Ionesi E. (2017). Inhibition of miR-208b improves cardiac function in titin-based dilated cardiomyopathy. Int. J. Cardiol. 230, 634–641. 10.1016/j.ijcard.2016.12.171 28065693

[B54] Zhuang S. Fu Y. Li J. Li M. Hu X. Zhu J. (2020). MicroRNA-375 overexpression disrupts cardiac development of Zebrafish (*Danio rerio*) by targeting notch2. Protoplasma 257, 1309–1318. 10.1007/s00709-020-01490-4 32468186

[B55] Zou J. F. Wu X. N. Shi R. H. Sun Y. Q. Qin F. J. Yang Y. M. (2020). Inhibition of microRNA-184 reduces H2O2-mediated cardiomyocyte injury via targeting FBXO28. Eur. Rev. Med. Pharmacol. Sci. 24, 11251–11258. 10.26355/eurrev_202011_23614 33215444

[B56] Zou X. Zhu D. Zhang H. Zhang S. Zhou X. He X. (2020). MicroRNA expression profiling analysis in serum for nasopharyngeal carcinoma diagnosis. Gene 727, 144243. 10.1016/j.gene.2019.144243 31743768

