# Supplementary figures and images for: Circulating microRNA: Myocardium-derived prenatal biomarker of ventricular septal defects
Source: Front Genet. 2022 Aug 11;13:899034. doi: 10.3389/fgene.2022.899034 (PMC9403759; doi:10.3389/fgene.2022.899034)

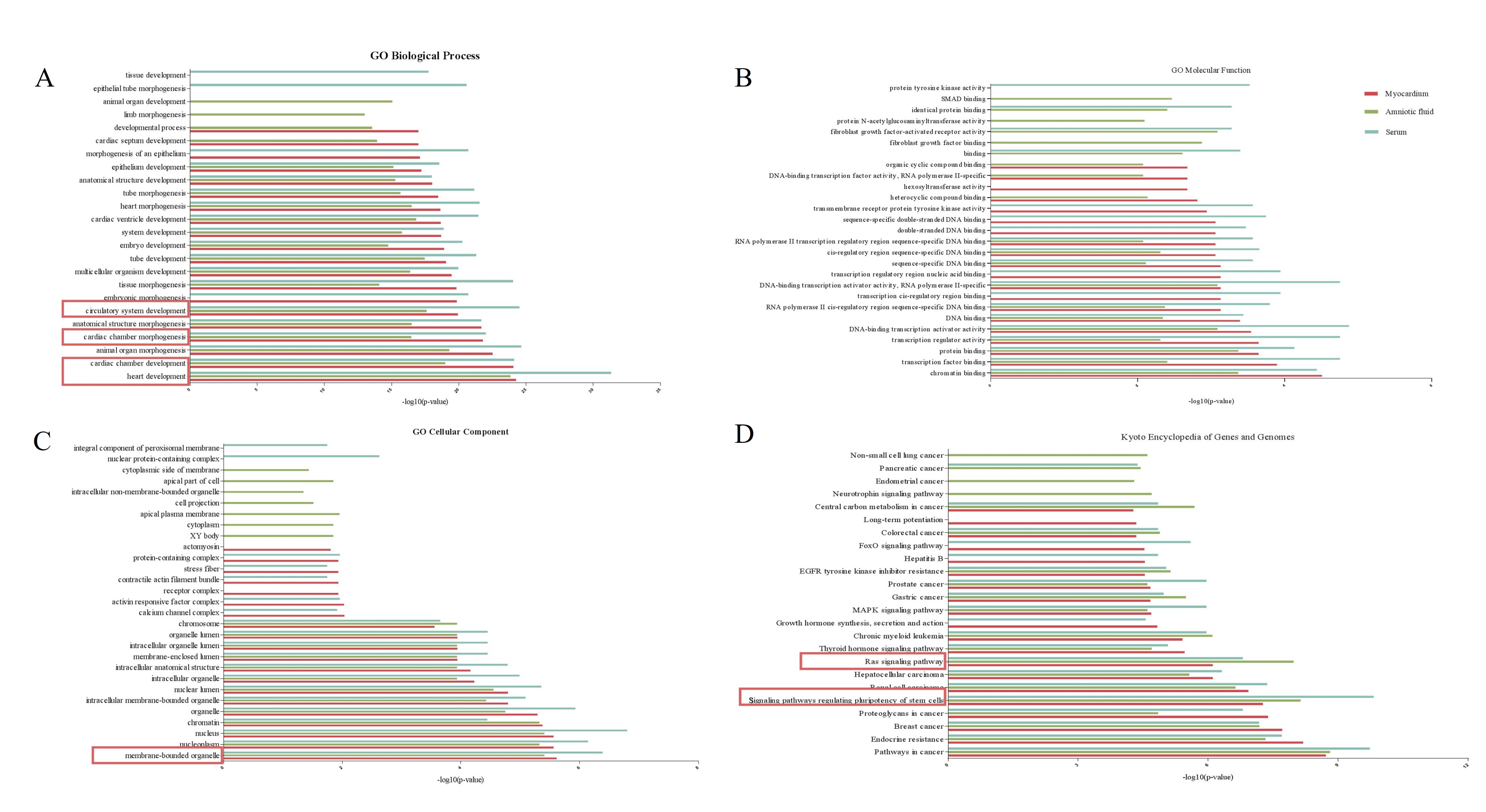

Supplement: Supplementary file 1 [file Image1.jpg]
