# Supplementary material for: Circulating microRNA: Myocardium-derived prenatal biomarker of ventricular septal defects
Source: Front Genet. 2022 Aug 11;13:899034. doi: 10.3389/fgene.2022.899034 (PMC9403759; doi:10.3389/fgene.2022.899034)
Supplement: Supplementary file 2 [file DataSheet1.docx]

**Supplement Tables**

**Table S1 Primer sequence of qRT–PCR**

| ID | RNA primer sequence (Forward) |
| --- | --- |
| rno-miR-1-3p | CGCCGTGGAATGTAAAGAAGTGTGTA |
| rno-miR-1b | GCGGCCTGGAATGTAAAGAAGTATGT |
| rno-miR-293-5p | CGCCACTCAAACTGTGTGACACTTT |
| rno-miR-3580-3p | CGGCCTGACTAGGGTAGTATGAGTAGA |
| rno-miR-206-3p | CCGTGGAATGTAAGGAAGTGTGTGG |
| rno-miR-142-3p | AACCGGTGTAGTGTTTCCTACT |
| rno-miR-497-3p | ATGCCAATCCAAACCACACTG |
| rno-miR-185-5p | AACCATGTGGAGAGAAAGGCA |
| rno-miR-874-5p | GTTAACATATCGGCCCCACG |
| rno-miR-96-5p | CTTTGGCACTAGCACATTTTTGCT |
| rno-miR-15b-5p | CGCTAGCAGCACATCATGGTTTACA |
| rno-miR-199a-5p | GCCCAGTGTTCAGACTACCTGTTC |
| rno-miR-122-5p | AACACGCTGGAGTGTGACAA |
| rno-miR-134-3p | AAGAATCTGTGGGCCACCTAG |
| rno-miR-184 | AACCGGTGGACGGAGAACT |
| rno-miR-129-5p | CTTTTTGCGGTCTGGGCTTGC |
| rno-miR-494-3p | CGTGAAACATACACGGGAAACCTCT |
| rno-miR-208b-3p | GCGGCCATAAGACGAACAAAAGGT |
| rno-miR-877 | CCACCATGGTAGAGGAGATGG |
| rno-miR-433-3p | AATCCGGAATCATGATGGGCT |
| hsa-miR-1-3p | GCGCCTGGAATGTAAAGAAGTATGTA |
| hsa-miR-15b-5p | AGTAGCAGCACATCATGGTTTACA |
| hsa-miR-206 | AGTGGAATGTAAGGAAGTGTGTGG |
| has-miR-184 | CGCGCTTATAATACAACCTGATAAGTG |

**Table S2 Myocardial tissue-derived DE-microRNAs in the VSD group**

| ID | Regulation | Fold Change | P-value | intersection with non-VSD group | ID | Regulation | Fold Change | P-value | intersection with non-VSD group |
| --- | --- | --- | --- | --- | --- | --- | --- | --- | --- |
| rno-miR-136-5p | up | 2.111 | 0.011 | NO | rno-miR-293-5p | down | 0.457 | 0.044 | NO |
| rno-miR-137-3p | up | 3.857 | 0.001 | NO | rno-miR-3064-5p | down | 0.458 | 0.016 | NO |
| rno-miR-146a-5p | up | 2.151 | 0.000 | NO | rno-miR-3473 | down | 0.356 | 0.000 | NO |
| rno-miR-19b-1-5p | up | 2.515 | 0.013 | NO | rno-miR-3580-3p | down | 0.124 | 0.009 | NO |
| rno-miR-203a-3p | up | 2.441 | 0.029 | NO | rno-miR-363-5p | down | 0.293 | 0.034 | NO |
| rno-miR-29b-3p | up | 2.272 | 0.002 | NO | rno-miR-741-3p | down | 0.301 | 0.048 | NO |
| rno-miR-301a-3p | up | 2.264 | 0.001 | NO | rno-miR-871-5p | down | 0.217 | 0.040 | NO |
| rno-miR-325-5p | up | 2.308 | 0.023 | NO | rno-miR-883-3p | down | 0.126 | 0.031 | NO |
| rno-miR-338-3p | up | 2.122 | 0.029 | NO | rno-miR-135b-3p | up | 8.012 | 0.001 | YES |
| rno-miR-376a-3p | up | 2.248 | 0.007 | NO | rno-miR-135b-5p | up | 6.667 | 0.001 | YES |
| rno-miR-384-3p | up | 2.832 | 0.017 | NO | rno-miR-190a-5p | up | 2.595 | 0.000 | YES |
| rno-miR-384-5p | up | 2.344 | 0.019 | NO | rno-miR-142-5p | down | 0.172 | 0.000 | YES |
| rno-miR-466c-5p | up | 2.026 | 0.002 | NO | rno-miR-144-3p | down | 0.165 | 0.000 | YES |
| rno-miR-497-3p | up | 10.827 | 0.019 | NO | rno-miR-144-5p | down | 0.193 | 0.000 | YES |
| rno-miR-497-5p | up | 2.098 | 0.004 | NO | rno-miR-191b | down | 0.288 | 0.042 | YES |
| rno-miR-539-3p | up | 2.199 | 0.024 | NO | rno-miR-193b-3p | down | 0.413 | 0.003 | YES |
| rno-miR-539-5p | up | 2.093 | 0.035 | NO | rno-miR-196b-5p | down | 0.289 | 0.040 | YES |
| rno-miR-7b | up | 4.196 | 0.000 | NO | rno-miR-206-3p | down | 0.142 | 0.001 | YES |
| rno-miR-874-5p | up | 3.436 | 0.011 | NO | rno-miR-3590-3p | down | 0.171 | 0.000 | YES |
| rno-miR-96-5p | up | 4.068 | 0.001 | NO | rno-miR-451-3p | down | 0.227 | 0.001 | YES |
| rno-miR-1-3p | down | 0.437 | 0.025 | NO | rno-miR-451-5p | down | 0.268 | 0.001 | YES |
| rno-miR-106b-3p | down | 0.430 | 0.003 | NO | rno-miR-486 | down | 0.199 | 0.000 | YES |
| rno-miR-142-3p | down | 0.287 | 0.001 | NO | rno-miR-511-5p | down | 0.332 | 0.004 | YES |
| rno-miR-1839-5p | down | 0.497 | 0.006 | NO | rno-miR-6329 | down | 0.337 | 0.000 | YES |
| rno-miR-185-5p | down | 0.349 | 0.000 | NO | rno-miR-743b-5p | down | 0.203 | 0.003 | YES |
| rno-miR-1956-3p | down | 0.147 | 0.004 | NO | rno-miR-871-3p | down | 0.159 | 0.003 | YES |
| rno-miR-1b | down | 0.491 | 0.027 | NO |  |  |  |  |  |

**Table S3 Myocardial tissue-derived DE-microRNAs in the non-VSD group**

| ID | Regulation | Fold Change | P-value | intersection with VSD group | ID | Regulation | Fold Change | P-value | intersection with VSD group |
| --- | --- | --- | --- | --- | --- | --- | --- | --- | --- |
| rno-miR-132-3p | up | 2.190 | 0.004 | NO | rno-miR-135b-5p | up | 3.945 | 0.013 | YES |
| rno-miR-132-5p | up | 2.221 | 0.003 | NO | rno-miR-190a-5p | up | 2.153 | 0.002 | YES |
| rno-miR-196c-5p | up | 3.255 | 0.043 | NO | rno-miR-142-5p | down | 0.328 | 0.008 | YES |
| rno-miR-291a-3p | up | 3.063 | 0.005 | NO | rno-miR-144-3p | down | 0.409 | 0.036 | YES |
| rno-miR-295-3p | up | 3.142 | 0.003 | NO | rno-miR-144-5p | down | 0.247 | 0.001 | YES |
| rno-miR-547-3p | up | 2.355 | 0.004 | NO | rno-miR-191b | down | 0.222 | 0.028 | YES |
| rno-miR-124-5p | down | 0.388 | 0.047 | NO | rno-miR-193b-3p | down | 0.488 | 0.008 | YES |
| rno-miR-136-3p | down | 0.435 | 0.007 | NO | rno-miR-196b-5p | down | 0.267 | 0.037 | YES |
| rno-miR-183-3p | down | 0.427 | 0.048 | NO | rno-miR-206-3p | down | 0.169 | 0.007 | YES |
| rno-miR-341 | down | 0.492 | 0.019 | NO | rno-miR-3590-3p | down | 0.230 | 0.001 | YES |
| rno-miR-34b-5p | down | 0.465 | 0.028 | NO | rno-miR-451-3p | down | 0.238 | 0.002 | YES |
| rno-miR-34c-5p | down | 0.460 | 0.011 | NO | rno-miR-451-5p | down | 0.328 | 0.006 | YES |
| rno-miR-6321 | down | 0.034 | 0.002 | NO | rno-miR-486 | down | 0.311 | 0.002 | YES |
| rno-miR-743a-5p | down | 0.157 | 0.021 | NO | rno-miR-511-5p | down | 0.431 | 0.050 | YES |
| rno-miR-7a-5p | down | 0.443 | 0.003 | NO | rno-miR-6329 | down | 0.414 | 0.000 | YES |
| rno-miR-879-5p | down | 0.382 | 0.028 | NO | rno-miR-743b-5p | down | 0.314 | 0.041 | YES |
| rno-miR-135b-3p | up | 6.166 | 0.004 | YES | rno-miR-871-3p | down | 0.226 | 0.045 | YES |

**Table S4 Amniotic fluid-derived DE-microRNAs in the VSD group**

| ID | Regulation | Fold Change | P-value | intersection with non-VSD group | ID | Regulation | Fold Change | P-value | intersection with non-VSD group |
| --- | --- | --- | --- | --- | --- | --- | --- | --- | --- |
| rno-miR-122-5p | up | 6.758 | 0.000 | NO | rno-miR-376b-3p | down | 0.243 | 0.001 | NO |
| rno-miR-1306-5p | up | 2.099 | 0.021 | NO | rno-miR-376c-3p | down | 0.323 | 0.027 | NO |
| rno-miR-134-3p | up | 16.200 | 0.048 | NO | rno-miR-485-5p | down | 0.466 | 0.040 | NO |
| rno-miR-1-3p | up | 4.010 | 0.000 | NO | rno-miR-1224 | up | 6.771 | 0.004 | YES |
| rno-miR-15b-5p | up | 2.365 | 0.009 | NO | rno-miR-142-3p | up | 4.540 | 0.000 | YES |
| rno-miR-184 | up | 3.048 | 0.013 | NO | rno-miR-142-5p | up | 3.635 | 0.002 | YES |
| rno-miR-1b | up | 2.343 | 0.002 | NO | rno-miR-144-3p | up | 5.365 | 0.000 | YES |
| rno-miR-206-3p | up | 3.876 | 0.000 | NO | rno-miR-144-5p | up | 4.638 | 0.000 | YES |
| rno-miR-665 | up | 2.163 | 0.022 | NO | rno-miR-185-5p | up | 3.043 | 0.000 | YES |
| rno-miR-708-5p | up | 2.029 | 0.027 | NO | rno-miR-190a-5p | up | 2.799 | 0.014 | YES |
| rno-miR-136-3p | down | 0.421 | 0.009 | NO | rno-miR-25-5p | up | 7.639 | 0.010 | YES |
| rno-miR-1843b-5p | down | 0.027 | 0.040 | NO | rno-miR-292-5p | up | 2.632 | 0.006 | YES |
| rno-miR-199a-3p | down | 0.406 | 0.002 | NO | rno-miR-451-5p | up | 2.811 | 0.001 | YES |
| rno-miR-199a-5p | down | 0.411 | 0.019 | NO | rno-miR-484 | up | 2.709 | 0.017 | YES |
| rno-miR-299a-5p | down | 0.166 | 0.038 | NO | rno-miR-486 | up | 2.122 | 0.001 | YES |
| rno-miR-300-3p | down | 0.363 | 0.000 | NO | rno-miR-382-3p | down | 0.134 | 0.000 | YES |
| rno-miR-369-3p | down | 0.407 | 0.011 | NO |  |  |  |  |  |

**Table S5 Amniotic fluid-derived DE-microRNAs in the non-VSD group**

| ID | Regulation | Fold Change | P-value | intersection with VSD group | ID | Regulation | Fold Change | P-value | intersection with VSD group |
| --- | --- | --- | --- | --- | --- | --- | --- | --- | --- |
| rno-miR-101a-3p | up | 3.513 | 0.004 | NO | rno-miR-92b-5p | up | 2.843 | 0.033 | NO |
| rno-miR-106b-5p | up | 3.438 | 0.020 | NO | rno-miR-9a-3p | up | 6.076 | 0.000 | NO |
| rno-miR-124-3p | up | 40.000 | 0.004 | NO | rno-miR-9a-5p | up | 3.323 | 0.003 | NO |
| rno-miR-1247-3p | up | 5.382 | 0.019 | NO | rno-let-7e-5p | down | 0.473 | 0.018 | NO |
| rno-miR-1247-5p | up | 2.102 | 0.020 | NO | rno-miR-196a-5p | down | 0.371 | 0.019 | NO |
| rno-miR-126a-3p | up | 2.833 | 0.000 | NO | rno-miR-27b-3p | down | 0.369 | 0.001 | NO |
| rno-miR-145-5p | up | 2.853 | 0.009 | NO | rno-miR-30a-3p | down | 0.362 | 0.048 | NO |
| rno-miR-147 | up | 40.400 | 0.003 | NO | rno-miR-30e-3p | down | 0.156 | 0.002 | NO |
| rno-miR-151-5p | up | 2.376 | 0.019 | NO | rno-miR-3553 | down | 0.495 | 0.033 | NO |
| rno-miR-181a-2-3p | up | 19.067 | 0.005 | NO | rno-miR-672-5p | down | 0.234 | 0.013 | NO |
| rno-miR-208b-3p | up | 2.405 | 0.050 | NO | rno-miR-98-5p | down | 0.330 | 0.018 | NO |
| rno-miR-292-3p | up | 20.667 | 0.011 | NO | rno-miR-1224 | up | 5.264 | 0.021 | YES |
| rno-miR-298-3p | up | 33.400 | 0.013 | NO | rno-miR-142-3p | up | 6.932 | 0.000 | YES |
| rno-miR-323-5p | up | 9.500 | 0.048 | NO | rno-miR-142-5p | up | 5.620 | 0.000 | YES |
| rno-miR-3557-5p | up | 2.819 | 0.001 | NO | rno-miR-144-3p | up | 8.979 | 0.000 | YES |
| rno-miR-363-3p | up | 4.069 | 0.001 | NO | rno-miR-144-5p | up | 8.025 | 0.000 | YES |
| rno-miR-363-5p | up | 81.900 | 0.000 | NO | rno-miR-185-5p | up | 4.181 | 0.000 | YES |
| rno-miR-423-3p | up | 2.035 | 0.009 | NO | rno-miR-190a-5p | up | 3.990 | 0.001 | YES |
| rno-miR-450a-5p | up | 2.354 | 0.015 | NO | rno-miR-25-5p | up | 6.235 | 0.042 | YES |
| rno-miR-483-5p | up | 2.022 | 0.001 | NO | rno-miR-292-5p | up | 2.787 | 0.002 | YES |
| rno-miR-544-3p | up | 20.700 | 0.040 | NO | rno-miR-451-5p | up | 5.580 | 0.000 | YES |
| rno-miR-743b-3p | up | 30.800 | 0.008 | NO | rno-miR-484 | up | 3.721 | 0.001 | YES |
| rno-miR-7a-5p | up | 2.428 | 0.008 | NO | rno-miR-486 | up | 2.208 | 0.000 | YES |
| rno-miR-874-3p | up | 9.729 | 0.008 | NO | rno-miR-382-3p | down | 0.374 | 0.029 | YES |

**Table S6 Maternal serum-derived DE-microRNAs in the VSD group**

| ID | Regulation | Fold Change | P-value | ID | Regulation | Fold Change | P-value |
| --- | --- | --- | --- | --- | --- | --- | --- |
| rno-miR-1188-5p | up | 2.130 | 0.001 | rno-miR-540-3p | up | 2.366 | 0.001 |
| rno-miR-129-5p | up | 2.232 | 0.037 | rno-miR-6216 | up | 2.055 | 0.014 |
| rno-miR-134-5p | up | 2.195 | 0.005 | rno-miR-665 | up | 2.453 | 0.018 |
| rno-miR-136-3p | up | 2.343 | 0.007 | rno-miR-743b-5p | up | 2.781 | 0.036 |
| rno-miR-206-3p | up | 2.043 | 0.021 | rno-miR-877 | up | 2.272 | 0.005 |
| rno-miR-211-5p | up | 9.850 | 0.008 | rno-miR-9a-3p | up | 6.148 | 0.038 |
| rno-miR-290 | up | 5.733 | 0.006 | rno-miR-147 | down | 0.410 | 0.031 |
| rno-miR-291a-5p | up | 5.855 | 0.000 | rno-miR-208a-3p | down | 0.196 | 0.005 |
| rno-miR-292-5p | up | 4.068 | 0.000 | rno-miR-208b-3p | down | 0.188 | 0.030 |
| rno-miR-293-5p | up | 3.069 | 0.010 | rno-miR-465-5p | down | 0.150 | 0.018 |
| rno-miR-3580-3p | up | 2.447 | 0.003 | rno-miR-488-3p | down | 0.101 | 0.000 |
| rno-miR-370-3p | up | 2.821 | 0.010 | rno-miR-490-3p | down | 0.455 | 0.036 |
| rno-miR-433-3p | up | 2.255 | 0.001 | rno-miR-873-3p | down | 0.087 | 0.027 |
| rno-miR-494-3p | up | 2.450 | 0.004 |  |  |  |  |

**Table S7 Hub genes of the VSD group**

| Heart tissue | | | | | | | Amniotic fluid | | | | | | | Serum | | | | | | |
| --- | --- | --- | --- | --- | --- | --- | --- | --- | --- | --- | --- | --- | --- | --- | --- | --- | --- | --- | --- | --- |
| MCC | DMNC | MNC | Degree | EPC | Closeness | Radiality | MCC | DMNC | MNC | Degree | EPC | Closeness | Radiality | MCC | DMNC | MNC | Degree | EPC | Closeness | Radiality |
| **Ptpn11** | Ptk2 | Notch1 | Notch1 | Notch1 | Notch1 | Notch1 | Kras | Fgfr2 | Kras | Notch1 | Ntrk3 | Notch1 | Notch1 | Kras | Fgfr3 | Notch1 | Notch1 | Pik3ca | Notch1 | Acta2 |
| **Kras** | Fgfr3 | **Kras** | **Kras** | Hras | Crebbp | Crebbp | Hras | Fgfr3 | Notch1 | Kras | Wt1 | Kras | Kras | Pik3ca | Fgfr2 | Kras | Kras | Hras | Kras | Notch1 |
| Hras | Ntrk3 | Hras | Hras | **Kras** | **Kras** | **Kras** | Pik3ca | Ntrk3 | Hras | Hras | Agt | Hras | Smad4 | Hras | Ntrk3 | Pik3ca | Pik3ca | Kras | Pik3ca | Kras |
| Pik3ca | Sos1 | Crebbp | Crebbp | Pik3ca | Hras | Smad4 | Ptpn11 | Shoc2 | Pik3ca | Pik3ca | Pbrm1 | Smad4 | Hras | **Ptpn11** | Sos1 | Hras | Hras | Notch1 | Acta2 | Pik3ca |
| Pik3r2 | Pik3r2 | Pik3ca | Pik3ca | **Ptpn11** | Pik3ca | Hras | Pik3r2 | Ntf3 | Smad4 | Smad4 | Tbx5 | Pik3ca | Ptpn11 | Pik3r2 | Pik3r2 | Crebbp | Crebbp | **Ptpn11** | Crebbp | Crebbp |
| Sos1 | **Ptpn11** | Smad4 | Gata4 | **Igf1r** | Gata4 | Gata4 | Sos1 | **Abl1** | Ptpn11 | Ptpn11 | **Cxcr4** | Ptpn11 | Pik3ca | Sos1 | Ptk2 | Smad4 | Smad4 | Smad4 | Smad4 | Smad4 |
| Ptk2 | Braf | **Ptpn11** | Smad4 | Crebbp | Smad4 | Pik3ca | Fgfr2 | Pik3r2 | **Map2k1** | **Map2k1** | **Map2k1** | **Map2k1** | **Map2k1** | Ptk2 | **Ptpn11** | Acta2 | Acta2 | **Map2k1** | Hras | **Ptpn11** |
| **Igf1r** | Cxcr4 | Gata4 | **Ptpn11** | Smad4 | **Ptpn11** | Sox2 | Fgfr1 | Sos1 | Sos1 | Gata4 | Gata4 | Gata4 | **Abl1** | Fgfr2 | Ntf3 | **Ptpn11** | **Ptpn11** | **Igf1r** | **Ptpn11** | Hras |
| **Map2k1** | Abl1 | **Igf1r** | **Igf1r** | **Map2k1** | **Map2k1** | Wt1 | Fgfr3 | Gnb1 | Gata4 | Sos1 | Braf | Fgfr1 | Fgfr1 | **Fgfr1** | Cxcr4 | Nkx2-5 | Nkx2-5 | Sos1 | Nkx2-5 | Wt1 |
| Abl1 | Rit1 | **Map2k1** | **Map2k1** | **Fgfr1** | **Igf1r** | **Map2k1** | **Abl1** | Braf | Pik3r2 | Pik3r2 | Gnb1 | **Abl1** | Wt1 | Fgfr3 | Braf | **Igf1r** | **Igf1r** | Crebbp | **Igf1r** | Edn1 |
| Notch1 | Gnb1 | Wt1 | Wt1 | Sos1 | Sox2 | **Fgfr1** | Notch1 | Ptpn11 | Wt1 | Fgfr1 | Jag1 | Wt1 | Gata4 | **Igf1r** | Abl1 | **Map2k1** | **Map2k1** | **Fgfr1** | Edn1 | Nkx2-5 |
| **Fgfr1** | Igf2 | Sos1 | Sos1 | Pik3r2 | Wt1 | **Ptpn11** | Smad4 | Fgfr1 | Fgfr1 | Wt1 | **Tek** | Agt | Agt | **Map2k1** | **Igf1r** | Sos1 | Sos1 | Tek | Wt1 | Sox2 |
| Gnb1 | Rras2 | **Fgfr1** | **Fgfr1** | Edn1 | **Fgfr1** | **Igf1r** | **Map2k1** | Rit1 | Agt | Agt | Ntf3 | Pik3r2 | **Cxcr4** | Notch1 | Gnb1 | Pik3r2 | Pik3r2 | Pik3r2 | Sox2 | **Fgfr1** |
| Cxcr4 | **Igf1r** | Prkaca | Prkaca | Gata4 | Jag1 | Gli2 | Ntf3 | **Cxcr4** | **Tek** | **Cxcr4** | Fgfr2 | **Cxcr4** | Pbrm1 | Smad4 | **Map2k1** | **Fgfr1** | **Fgfr1** | Acta2 | **Map2k1** | Tek |
| Smad4 | **Map2k1** | Pik3r2 | Edn1 | Ptk2 | Prkaca | Jag1 | Ntrk3 | Igf2 | **Abl1** | **Tek** | Hey2 | Pbrm1 | Pik3r2 | Cxcr4 | Igf2 | Prkaca | Prkaca | Edn1 | **Fgfr1** | Tgfb1 |
| Fgfr3 | Tek | Edn1 | Pik3r2 | Prkaca | Gli2 | Abl1 | Gnb1 | **Map2k1** | **Cxcr4** | **Abl1** | Notch2 | Jag1 | Jag1 | Abl1 | Rit1 | Wt1 | Edn1 | Ptk2 | Tek | **Igf1r** |
| Ntrk3 | Cacna1d | Jag1 | Sox2 | Wt1 | Edn1 | Cxcr4 | Igf2 | Prkaca | Jag1 | Ntrk3 | Prkaca | Sos1 | Fgfr3 | Ntf3 | Rras2 | Edn1 | Tek | Rras2 | Jag1 | Agt |
| Braf | **Fgfr1** | Gata6 | Rras2 | Cxcr4 | Cxcr4 | Gata6 | **Tek** | **Tek** | Ntf3 | Gnb1 | Dnmt3a | **Tek** | Fgfr2 | Ntrk3 | **Fgfr1** | Agt | Wt1 | Agt | Agt | **Map2k1** |

**Table S7(continued)**

| Heart tissue | | | | | | | Amniotic fluid | | | | | | | Serum | | | | | | |
| --- | --- | --- | --- | --- | --- | --- | --- | --- | --- | --- | --- | --- | --- | --- | --- | --- | --- | --- | --- | --- |
| MCC | DMNC | MNC | Degree | EPC | Closeness | Radiality | MCC | DMNC | MNC | Degree | EPC | Closeness | Radiality | MCC | DMNC | MNC | Degree | EPC | Closeness | Radiality |
| Tek | Myocd | Ptk2 | Jag1 | Abl1 | Abl1 | Pbrm1 | **Cxcr4** | Dll4 | Fgfr2 | Jag1 | **Abl1** | Fgfr3 | **Tek** | Gnb1 | Tek | Tek | Ptk2 | Fgfr2 | Tgfb1 | Jag1 |
| Prkaca | **Kras** | Sox2 | Cxcr4 | Rras2 | Gata6 | Edn1 | Braf | Pik3ca | Igf2 | Ntf3 | Sos1 | Fgfr2 | Igf2 | Agt | Myocd | Jag1 | Agt | Wt1 | Ptk2 | Pbrm1 |

**Table S8 Comparison of rat and human amniotic fluid-derived DE-microRNA sequences**

| ID | Sequence | | |
| --- | --- | --- | --- |
| rno-miR-1-3p | UGGAAUGUAAAGAAGUGUGUAU | | |
| hsa-miR-1-3p | UGGAAUGUAAAGAAGUAUGUAU | | |
|  |  |  |  |
| rno-miR-206-3p | UGGAAUGUAAGGAAGUGUGUGG | | |
| hsa-miR-206 | UGGAAUGUAAGGAAGUGUGUGG | | |
|  |  |  |  |
| rno-miR-15b-5p | UAGCAGCACAUCAUGGUUUACA | | |
| hsa-miR-15b-5p | UAGCAGCACAUCAUGGUUUACA | | |
|  |  |  |  |
| rno-miR-184 | TGGACGGAGAACTGATAAGGGT | | |
| hsa-miR-184 | TGGACGGAGAACTGATAAGGGT | | |
